# Radiation-induced liver disease mimicking liver metastasis after low-dose hepatic irradiation during radiotherapy for gastric mucosa-assisted lymphoid tissue lymphoma: A case report

**DOI:** 10.1097/MD.0000000000039191

**Published:** 2024-08-23

**Authors:** Hyeli Park, Sun Young Lee

**Affiliations:** a Department of Radiation Oncology, Presbyterian Medical Center, Jeonju, Jeonbuk, Republic of Korea; b Department of Medicine, Jeonbuk National University, Jeonju, Jeonbuk, Republic of Korea; c Department of Radiation Oncology, Jeonbuk National University Hospital-Jeonbuk National University Medical School, Jeonju, Jeonbuk, Republic of Korea; d Research Institute of Clinical Medicine of Jeonbuk National University-Biomedical Research Institute of Jeonbuk National University Hospital, Jeonju, Jeonbuk, Republic of Korea.

**Keywords:** liver disease, radiation tolerance, radiotherapy

## Abstract

**Rationale:**

Radiation-induced liver disease (RILD) is an established complication of hepatic irradiation that is typically reported in patients receiving high-dose radiotherapy for hepatocellular carcinoma or liver metastases. However, RILD can also occur after unintentional low-dose liver exposure during radiotherapy for other gastrointestinal malignancies when careful precautions are not taken.

**Patient concerns:**

We report the case of a 44-year-old woman with gastric mucosa-associated lymphoid tissue lymphoma who underwent salvage radiotherapy administered to the entire stomach. One month after completing this radiotherapy, computed tomography and magnetic resonance imaging of the patient’s abdomen revealed a 4 cm lesion in the left lateral liver segment, suggestive of metastasis.

**Diagnoses:**

An ultrasound-guided biopsy was performed, and the histopathological findings were consistent with those of RILD.

**Interventions:**

Conservative management was pursued with close monitoring of liver function tests.

**Outcomes:**

The patient’s imaging findings and liver enzyme levels normalized approximately 3 months after the initial diagnosis.

**Lessons:**

This case highlights the importance of considering RILD in the differential diagnosis of new hepatic lesions detected after radiotherapy, even in patients with low-dose liver exposure within generally acceptable limits. Careful correlation with the radiotherapy plan is crucial to avoid misdiagnosing RILD as metastatic disease and to guide appropriate management.

## 1. Introduction

Radiation-induced liver disease (RILD) is a well-recognized complication associated with hepatic irradiation.^[1]^ RILD is generally categorized into two categories: classic and nonclassic forms.^[2]^ Classic RILD, which typically develops after whole-liver irradiation, is characterized by ascites, analgesic hepatomegaly, thrombocytopenia, fatigue, abdominal pain, and elevated liver enzymes, especially alkaline phosphatase, with normal bilirubin and transaminase levels.^[3]^ In contrast, nonclassic RILD usually occurs after partial irradiation and is differentiated from classic RILD by elevated serum transaminases (to more than 5 times the upper limit of normal) and jaundice.^[4]^ With the increasing use of radiotherapeutic techniques that limit irradiation to partial liver volumes, nonclassic RILD has become more prevalent.^[2]^ RILD is commonly reported in patients who receive radiotherapy for primary and secondary liver malignancies. However, RILD can also occur after incidental liver exposure during abdominal irradiation for other malignancies.^[5,6]^ The clinical manifestations of radiation-induced liver toxicity can range from asymptomatic cases to liver failure.^[7]^

## 2. Case report

**Figure 1.**
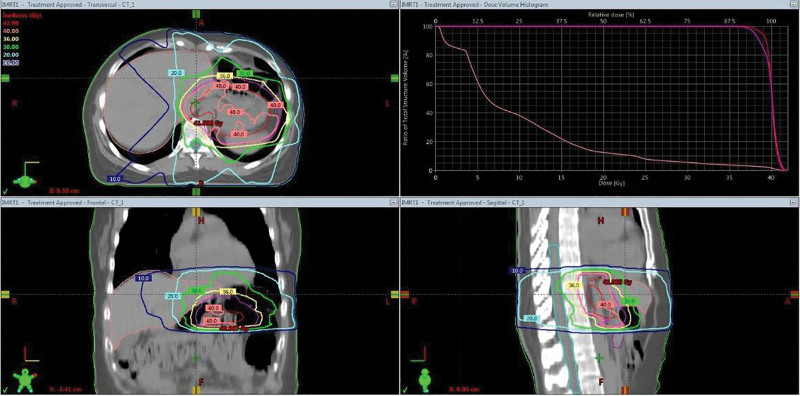
Planning image of the gastric MALT lymphoma. MALT = mucosa-associated lymphoid tissue.

A 44-year-old Asian woman was diagnosed with *Helicobacter pylori*-positive stage, that is, gastric mucosa-associated lymphoid tissue (MALT) lymphoma in February 2019. Despite undergoing *H pylori* eradication therapy, she had a residual gastric MALT lymphoma. Radiotherapy was administered to her entire stomach to treat this residual gastric MALT lymphoma. A total radiation dose of 40 Gy was delivered in 20 fractions over 4 weeks (5 d/wk), using intensity-modulated radiotherapy (IMRT), until October 2019 (Fig. 1). The gross target volume (GTV) was defined as the whole stomach, excluding regional lymph node areas. Irradiation was delivered after a fasting period of at least 9 hours from midnight onward. The GTV was expanded by 10 mm to create the clinical target volume (CTV), and the CTV was then expanded by an additional 10 mm to create the planning target volume. The radiotherapy dose was delivered using 6 MV photons in a linear accelerator (TB4072, Varian Medical Systems Inc., Palo Alto). The mean liver dose was 9.16 Gy, and the maximum liver dose was 40.47 Gy. The patient did not have any underlying liver disease.

**Figure 2.**
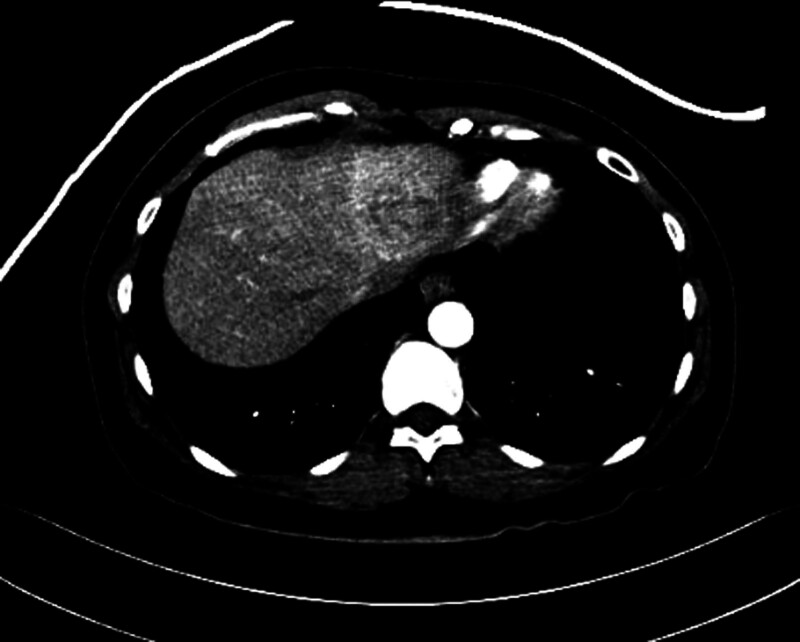
Abdominal and pelvic CT 1 mo after radiotherapy. Abdominal computed tomography revealed a 4 cm ill-defined, heterogeneously enhancing lesion in the superior portion of the left liver, suggestive of metastasis. CT = computed tomography.

**Figure 3.**
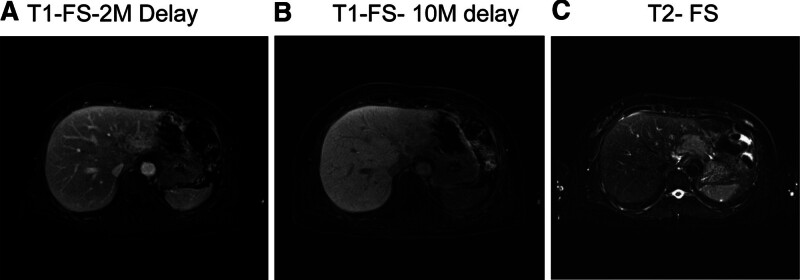
MRI performed 1 mo after the completion of radiation therapy. MRI revealed an irregularly shaped mass in the left lateral segment of the liver exhibiting high signal intensity on T2-weighted images and subtle enhancement. These imaging features suggested the involvement of lymphoma in the left lateral hepatic segment. FS = fat suppressed imaging, M = minutes, MRI = magnetic resonance imaging.

**Figure 4.**
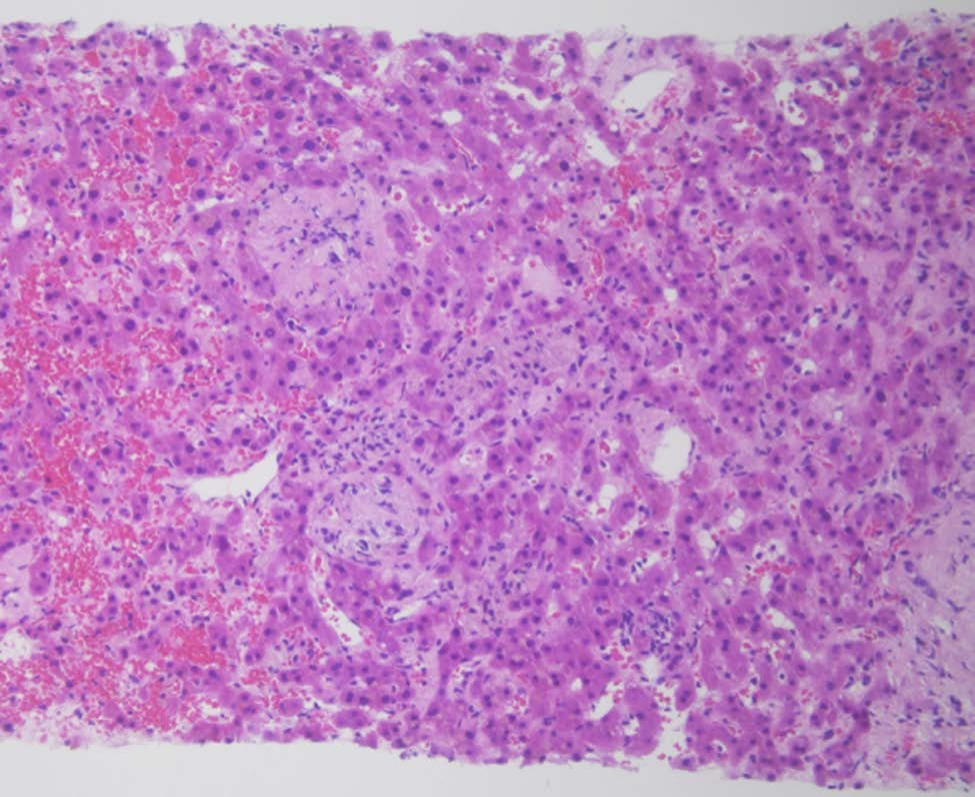
Histologic features of liver biopsies. Hematoxylin and Eosin (HE) staining, original magnification: 200×. Histopathological examination of the liver biopsy specimen revealed sinusoidal denudation, sinusoidal dilatation, and hemorrhage. Additionally, there was evidence of zone 3 (centrilobular) fibrosis and occlusion of the central vein. The liver tissue exhibited a characteristic sinusoidal and endothelial cell injury pattern.

In November 2019, 1 month after completing radiotherapy, the patient visited the emergency room due to acute abdominal pain. She had direct tenderness in her abdomen. Laboratory evaluation revealed elevated aspartate aminotransferase (AST) at 38 U/L and alanine aminotransferase (ALT) at 77 U/L (normal ranges: 0–32 U/L and 0–33 U/L, respectively). The alkaline phosphatase activity was within the normal range at 58 U/L (normal: 35–104 U/L; Table 1). Abdominal computed tomography (CT; Fig. 2) revealed a 4 cm enhancing lesion suggestive of metastasis in the left lateral segment of the liver. Magnetic resonance imaging (MRI) also suggested involvement in the left lateral segment of the liver (Fig. 3). An ultrasound-guided biopsy was performed for pathologic diagnosis. Histopathological examination of the liver biopsy specimen revealed sinusoidal denudation, lymphocytic infiltration, hemorrhage, and centrilobular fibrosis, consistent with the diagnosis of RILD (Fig. 4). These findings confirmed RILD rather than metastatic lymphoma, which was initially suspected based on the imaging findings. Upon fusion of the radiotherapy plan with postradiotherapy imaging, the 26 Gy isodose line correlated with the location of the hepatic lesion.

**Table 1:** Liver function test.

	2019-11-09	2019-11-21	2019-12-2	2020-3-16
ALP (normal ranges: 35–104 IU/L)	58	87	72	44
AST (normal ranges: 0–32 U/L)	38	40	41	21
ALT (normal ranges: 0–33 U/L)	77	59	45	22
T. bili (normal ranges: 0.2–1.2 mg/dL)	0.62	0.49	0.61	0.61

ALP = alkaline phosphatase, ALT = alanine aminotransferase, AST = aspartate aminotransferase, T. bili = total bilirubin.

**Figure 5.**
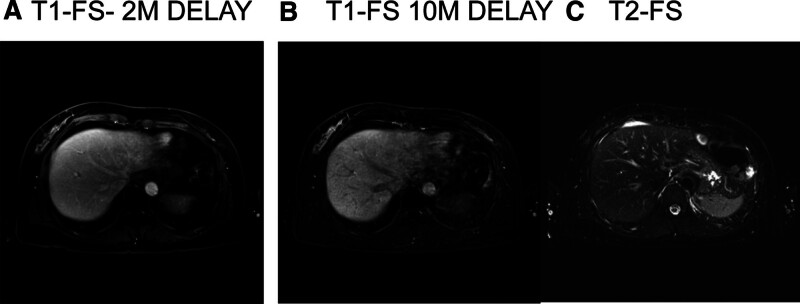
Follow-up MRI performed 2 mo after the radiation-induced liver disease diagnosis. FS = fat suppressed imaging, M = minutes, MRI = magnetic resonance imaging.

For the management of RILD, conservative treatment was pursued with close monitoring of liver function tests. The patient received supportive care including hepatoprotective agent containing ursodeoxycholic acid and biphenyl dimethyl dicarboxylate. Corticosteroids or immunosuppressive agents were not administered. On follow-up imaging, including MRI performed 2 months after the initial diagnosis of RILD, there was a decrease in the extent of the previously identified hepatic lesion in the left lateral lobe on the hepatobiliary phase indicating improved radiation-induced changes. On T2-weighted imaging, the lesion was no longer visible (Fig. 5). The patient’s elevated AST and ALT levels normalized after 4 months (Table 1). No residual lymphoma or hepatic lesions were detected on esophagogastroduodenoscopy and abdominal CT scan during the subsequent follow-up period until February 2024.

## 3. Discussion

The incidence of RILD ranges from 5% to 44%, depending on radiotherapy-related factors such as the radiation dose, irradiated liver volume, fraction size, and mean normal liver dose, as well as clinical factors such as the presence of primary hepatocellular carcinoma, history of hepatotoxic chemotherapy, and severity of hepatic cirrhosis.^[8–10]^ Various dosimetric parameters have been suggested to estimate the possibility of RILD. A whole-liver dose of <30 Gy in standard fractionation is generally considered safe. For stereotactic body radiation therapy (SBRT), it is recommended to maintain a minimum volume of 700 cc below 15 Gy.^[11]^ Liang et al^[12]^ proposed V20 (normal liver volume receiving >20 Gy) as a predictive indicator for RILD. Additionally, a nomogram has been developed to predict the risk of nonclassic RILD.^[13]^ The incidence of RILD has been decreasing due to advancements in radiotherapy technology and improved understanding of risk factors.^[7,13,14]^

Classic RILD typically manifests 2 weeks to 4 months after completing radiotherapy.^[2,7,15]^ The pathognomonic manifestation associated with classic RILD is veno-occlusive disease (VOD). Radiotherapy damages radiosensitive endothelial cells, leading to subsequent fibrin deposition and trapping of erythrocytes.^[16]^ These erythrocytes can occlude the central vein in the hepatic lobules.^[17]^ The resulting retrograde congestion and decreased oxygen supply to the central zone cause hepatocyte necrosis.^[10]^ Aside from injury to the endothelial cell lining, patients exhibiting severe congestive manifestations of typical RILD demonstrate activation of hepatic stellate cells.^[18]^ The onset of nonclassic RILD usually occurs 1 to 12 weeks after radiotherapy.^[7]^ Nonclassic RILD is often accompanied by underlying liver disease, and its pathogenesis is not entirely clear. However, the loss of regenerating hepatocytes has been suggested as a potential mechanism. Additionally, hepatic sinusoidal endothelial death and hepatic stellate cell activation have been reported in nonclassic RILD patients. Such pathological changes could represent a consequential effect of radiation-induced mitotic disruption in hepatocytes undergoing regenerative division.^[2,3,17]^

In the present case, the patient developed RILD one month after completing radiotherapy, which aligns with the typical onset timeline for RILD. Moreover, her biopsy findings were consistent with the pathological features of classic RILD. However, the characteristic symptomatic hallmarks of classic RILD, including ascites, anicteric hepatomegaly, and elevated alkaline phosphatase, were notably absent, with only mild elevations in AST and ALT detected.

After radiotherapy, CT parenchymal changes often start 1 to 3 months after irradiation, peak at 1 to 6 months and resolve after 9 months.^[4,19–21]^ In the acute phase (1–3 months), CT scans may show low attenuation in the irradiated area on precontrast images and arterial hyperenhancement.^[4,7,16,19]^ Hypoenhancement in the portal venous phase persists, and increased delayed enhancement is observed in the subacute phase (3–6 months). In the chronic phase, the enhancement pattern typically normalizes.^[4,16]^ VOD-induced parenchymal changes, including fibrotic, hemorrhagic or necrotic changes, cause hepatic edema or congestion.^[7]^ This phenomenon contributes to the hypoenhancement observed in the irradiated area. In the case of preserved hepatic function, hepatic vein occlusion induces drainage from the portal vein and a compensatory increase in inflow from the hepatic artery. This mechanism explains the hyperdensity observed in the arterial phase on dynamic CT and MRI.^[7,20]^ Disturbed venous drainage causes contrast media to concentrate in dilated hepatic sinusoids and remain in the interstitium of hypertrophied fibrous tissue, leading to delayed phase enhancement.^[22]^

The reported threshold doses for observing density changes on follow-up CT vary, ranging from 35 to 45 Gy delivered in conventional fractionation to 26 to 35 Gy after SBRT.^[23–27]^

On MRI, T1 hypointensity and T2 hyperintensity can be observed due to focal edema. On contrast-enhanced dynamic MR images of the liver, the early arterial enhancement pattern is commonly seen to continue into delayed phases. Hyperintensity on diffusion-weighted imaging and a low apparent diffusion coefficient are also exhibited in the radiation zone.^[7]^

In the present case, the imaging findings of an enhancing lesion in the left lateral segment of the liver on CT and MRI one month after radiotherapy aligned with the expected appearance of RILD in the acute phase, occurring within the region that received approximately 26 Gy in conventional fractionation.

Kimura et al^[20]^ reported three types of dynamic CT findings of RILD following SBRT, which changed over time and were influenced by the patient’s Child–Pugh class. The imaging features of RILD are affected by the underlying pathologic changes and recovery induced by hemodynamic alterations and hepatic fibrosis.^[7]^ These factors contribute to the nonspecific and varied imaging findings of RILD, often making it challenging to differentiate RILD from other hepatic pathologies, such as metastatic lesions. In the era of 2D radiotherapy, attenuation differences bordering straight lines within the liver have been observed.^[28]^ However, with the advent of 3-dimensional radiotherapy techniques, including IMRT and SBRT, the imaging manifestations of radiation-induced liver changes have become more complex, further complicating the distinction between treatment effects and potential metastatic disease. CT and MRI may reveal hepatic lesions with variable enhancement patterns, mimicking neoplastic lesions. Furthermore, the onset of RILD can occur weeks to months after radiotherapy, further complicating the diagnostic process. In the present case, the imaging findings of an enhancing lesion in the left lateral segment of the liver on CT and MRI one month after radiotherapy initially raised suspicion for metastatic disease, necessitating histopathological confirmation to distinguish RILD from other potential etiologies.

Diagnostic imaging plays a crucial role in the evaluation of suspected RILD; however, the imaging findings can be nonspecific and overlap with other hepatic pathologies, including metastases.^[3]^ In addition to conventional CT and MRI, various functional MRI modalities employing liver-specific contrast agents are under active investigation as potential tools to detect radiation-induced liver damage.

Superparamagnetic iron oxide (SPIO)-enhanced T2-weighted gradient echo imaging may help detect early subclinical RILD reflecting Kupffer cell damage.^[29]^ Gadolinium ethoxybenzyl diethylenetriamine pentaacetic acid (Gd-EOB-DTPA)-enhanced magnetic resonance imaging (EOB-MRI) has also been suggested as a tool for detecting radiation injury after 1 to 4 months. Gd-EOB-DTPA is a hepatocyte-specific contrast agent that is mainly excreted through the bile ducts.^[30]^ EOB-MRI can detect liver damage using decreased uptake areas of Gd-EOB-DTPA.^[2,31]^ The reported thresholds for decreased Gd-EOB-DTPA uptake are 24 to 29 Gy and 29 to 35 Gy for the equivalent dose in 2 Gy fractions and the biologically effective dose, respectively.^[32]^ Sun et al^[31]^ reported that a radiation isodose range of 30 to 46 Gy corresponded to a decreased area of gadoxetic acid uptake. MRI employing gadobenate dimeglumine (Gd-BOPTA) has also been suggested as a tool to detect RILD.^[33]^

Functional liver imaging using 99^M^ Tc-sulfur colloid single-photon emission computed tomography (SPECT-CT) has been used to evaluate RILD using changes in isotope uptake. This modality has also been used to avoid high-functioning areas for radiotherapy planning.^[2,23]^

Misdiagnosing RILD as a metastatic disease can have significant clinical implications, potentially leading to unnecessary interventions or treatments based on an incorrect assumption. Therefore, it is crucial for clinicians to maintain a high index of suspicion for RILD in patients who have undergone hepatic irradiation and develop new hepatic lesions during follow-up. Conventional imaging techniques (CT and MRI) revealed signs of a potential metastatic lesion in this patient. This necessitated obtaining a biopsy and histopathological examination to confirm the diagnosis of RILD.

As functional imaging techniques continue to evolve, they may aid in the early detection and monitoring of RILD, facilitating timely intervention and management. For patients who have risk factors precluding biopsy, functional imaging or liver-specific imaging studies could help distinguish liver injury from disease progression.

This case report highlights the importance of considering RILD in the differential diagnosis of new hepatic lesions detected after radiotherapy, even when the imaging findings may initially suggest metastatic disease. Although advances in radiotherapy techniques, such as IMRT, have improved the ability to limit hepatic exposure, partial liver irradiation can still lead to focal liver injury, as observed in this case. Collaboration among radiation oncologists, radiologists, and pathologists is crucial for accurate diagnosis and appropriate management of RILD, as well as for developing strategies to minimize the risk of this potential complication during radiotherapy. This case also underscores the importance of making continuous efforts to reduce the normal organ dose to as low as reasonably achievable, even in cases with an acceptable dose distribution. In this patient, the focal liver injury caused by low-dose irradiation resolved without any sequelae with conservative treatments. Moreover, the severity of the imaging findings was not directly correlated with the clinical severity. She had only mild elevations of AST and ALT with a normal ALP level, which did not meet the criteria for classic or nonclassical RILD.

This study has some limitations. As this is a single case report, we cannot determine the risk factors for RILD occurrence even at low hepatic radiation doses. Additionally, the accurate diagnosis of RILD still requires invasive biopsy to guide appropriate management, a topic that warrants further investigation.

## 4. Conclusion

Various normal organ tolerance parameters and adjusted clinical factors are used to estimate the acceptable radiation dose and distribution to prevent RILD. However, even generally safe doses can cause changes in normal organs. A multidisciplinary approach for both diagnosis and treatment can help reduce misdiagnosis and facilitate proper management.

## Author contributions

**Conceptualization:** Hyeli Park.

**Data curation:** Sun Young Lee.

**Supervision:** Sun Young Lee.

**Writing – original draft:** Hyeli Park.

**Writing – review & editing:** Sun Young Lee.
